# Qualitative Research to Design Sustainable Community-Based Surveillance for Rabies in Northern Australia and Papua New Guinea

**DOI:** 10.3389/fvets.2017.00019

**Published:** 2017-02-22

**Authors:** Victoria J. Brookes, Emma Kennedy, Phillipa Dhagapan, Michael P. Ward

**Affiliations:** ^1^School of Veterinary Science, University of Sydney, Sydney, NSW, Australia; ^2^Animal Management Program, East Arnhem Regional Council, Nhulunbuy, NT, Australia

**Keywords:** rabies, canine, dog, participatory epidemiology, qualitative, interviews, surveillance, one health

## Abstract

Given the proximity and recent spread of rabies in Indonesia, effective rabies surveillance in dogs is a priority in Northern Australia and Papua New Guinea (PNG). Reporting of potential cases requires community engagement; therefore, the value and acceptability of such a system is critical to ensure sustainable surveillance. We used qualitative research methods to identify factors that influence the acceptability and value of community-based rabies surveillance. Thirty-two semi-structured interviews were conducted with informants in 16 communities in East Arnhem, the Northern Peninsula Area, the Torres Strait in Australia, and in Western Province, PNG. Thematic analysis identified common themes including the importance of verbal communication, particularly *via* radio, community meetings, and direct conversation. We also found that dogs have high value to community members through connection to culture, economic (especially hunting), and companionship. The greatest barrier to the reporting of sick dogs was insufficient veterinary services and the subsequent lack of treatment response. In some regions, acceptance that sick dogs are a normal daily occurrence and lack of trust of authorities were also barriers to reporting. The findings from this study will be used to design sustainable rabies surveillance in Northern Australia and PNG by utilizing traditional communication channels and building on existing and valued animal-management services. The methods and findings of this study complement previous quantitative research, so as to target surveillance to high-risk areas within these regions.

## Introduction

After almost a century of endemic rabies infection in the Greater Sunda Islands in Indonesia (Sumatra, Java, Borneo, and Sulawesi), canine-rabies has spread to historically free islands in the east during the previous two decades (1, 2). The Oceanic region is free of canine-rabies, but the most recently infected Indonesia islands—the southern Maluku Islands—are approximately 1,000 km from Papua New Guinea (PNG), the Torres Strait, and Queensland, Australia, and only 300 km from the coastline of the Northern Territory, Australia. While the drivers of the recent spread of rabies in Indonesia are unknown, infection of new islands has been attributed to movement of rabies-infected dogs associated with human activities such as fishing and visiting relatives (3). Sea trade routes and cultural links exist between people in the south-east Asian and Oceanic regions and although international regulations restrict the movement of dogs, risk assessments indicate that the probability of unregulated movement—and thus the entry of rabies-infected dogs—from Indonesia into Australia and PNG is not negligible (4).

The global burden of rabies is high—it is estimated that tens of thousands of human deaths occur annually, the majority of which are caused by bites from infected dogs (5). Dogs are abundant in Aboriginal and Torres Strait Islander (Indigenous) communities in both Australia and PNG. Although owned, they are usually allowed to roam freely (6). Additionally—and in contrast to other regions of the world in which rabies is endemic—Australia and PNG have feral and wild dog populations (7). Rabies is challenging to eliminate from dog populations (5, 8, 9); endemicity in either the domestic or wild dog populations in Australia and PNG could have devastating, long-term impacts on both human and animal health in these countries. Therefore, timely detection of a rabies incursion in PNG or Northern Australia is important to increase the probability of elimination and prevent human deaths.

A global framework for elimination of canine-rabies was recently jointly proposed by the World Health Organization, World Organisation for Animal Health (OIE), Food and Agriculture Organization of the United Nations, and Global Alliance for Rabies Control (10). Recommendations included a One Health approach requiring sustained resources, understanding of socio-cultural contexts, technical capacity, and organizational and political support to support elimination efforts. Effective rabies surveillance in dogs is an essential component of elimination efforts, required to facilitate control measures and prevent human deaths. The importance of surveillance was illustrated recently during an outbreak in Malaysia (11). An effective surveillance system needs to have high positive predictive value and timely detection. To achieve this, methods such as risk assessment and evaluation of diagnostic tests have been used to focus resources to high-risk pathways and address requirements for rapid diagnosis. However, a surveillance system must also be sustainable to ensure that collected data are of high quality with comprehensive geographic and temporal coverage. Key attributes of sustainable systems—for example, reliability, flexibility, and simplicity—are more difficult to incorporate into surveillance system design because they require an understanding of the acceptability and value of the surveillance to the individuals required to participate in the system. In the case of rabies surveillance, community participation is critical for the reporting of suspect cases; therefore, community acceptability and value of rabies surveillance is essential. This was demonstrated in a trial of community-based rabies surveillance in Kenya, in which high community engagement led to an increased rate of case detection (12).

Qualitative research methods such as ethnographic or interview-based studies can provide insights about the human-driven contexts and mechanisms that lead to, or influence, particular actions and outcomes (13). These methods can complement the findings of quantitative research such as observational and experimental studies and are now used widely in medical research to promote delivery of health services (14–16). In the context of animal health, informal interviews are included in the repertoire of methods used in the discipline of participatory epidemiology (17) and have been used to identify production constraints, disease impacts, and feasible control strategies for endemic diseases (18–20). These methods have also been used recently in the context of biosecurity research to evaluate the acceptability of existing surveillance systems using focus group discussions, interviews, and visualization approaches (21–23).

The objective of the current study was to use qualitative research methods to identify factors that will influence the acceptability and value of community-based rabies surveillance in PNG and Northern Australia. We aim to use the findings from this study to enhance existing non-specific surveillance and plan targeted, community-based, sustainable canine-rabies surveillance in PNG and Northern Australia. We also evaluate the use of qualitative research in this context.

## Materials and Methods

### Overview

The core research for this study involved informal, semi-structured interviews, which were conducted with individuals or small groups (two to six participants) of informants in their homes or workplaces. Qualitative analysis of transcripts identified themes relevant to the design of sustainable surveillance for canine-rabies. The procedures used in this study were approved by the Human Research Ethics Committee of The University of Sydney (project number 2016/192).

### Selection of Communities

**Figure 1:**
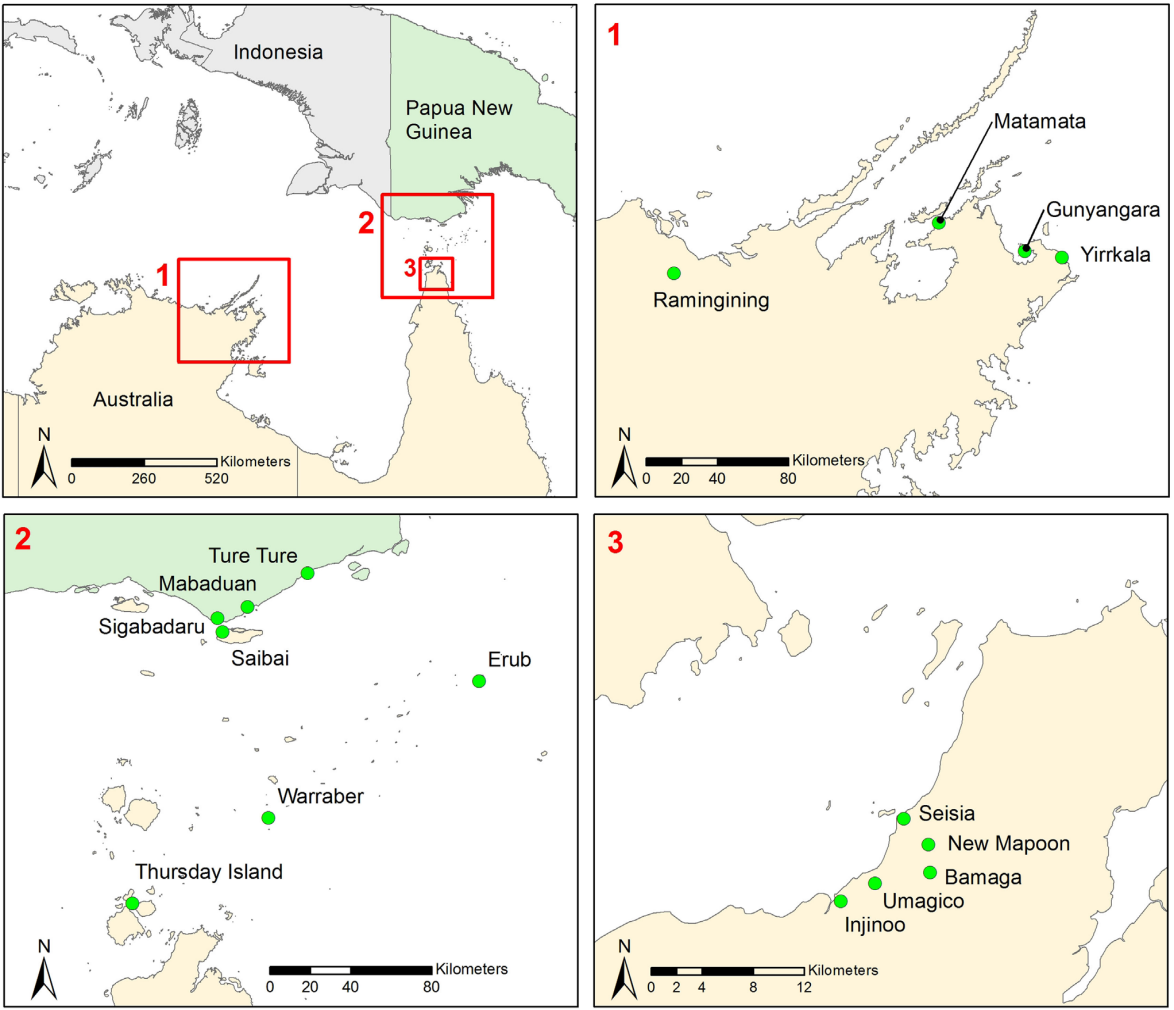
**Map showing location of communities included in a study to identify factors that will influence the acceptability and value of community-based rabies surveillance in Northern Australia and Papua New Guinea**.

The target population was residents of Indigenous communities in Northern Australia, the Torres Strait, and coastal Western Province, PNG. Sixteen communities were selected in East Arnhem in the Northern Territory, the Northern Peninsula Area (NPA) in QLD, the Torres Strait, and coastal Western Province in PNG (Figure 1). These regions have been identified to be at high risk of rabies incursion relative to other regions in PNG and Northern Australia in previous risk assessments [(4), B. Cookson, personal communication].

Demographic data about the Australian study regions were obtained from the Australian Bureau of Statistics, Census of Population and Housing 2011.^1^ Population density was estimated to be 0.32, 2.23, and 9.5 people/km^2^ in East Arnhem, the NPA, and the Torres Strait, respectively. The mean size of Australian communities included in this study was 588 people [median 375 people, range estimated 15–2,614 (Mata Mata and Thursday Island, respectively)]. In East Arnhem, the NPA, the Torres Strait, and the Australian general population, 60, 80, 72, and 79% of the population completed high-school education (year 10 or equivalent), respectively. The foundation-year 10 school curriculum is consistent throughout Australia.

Detailed population demographics were not available for the study region in PNG. The population density of South Fly, PNG was estimated to be 1.9 people/km^2^. The estimated population size of Mabaduan was 2,000 people (Mabaduan village recorder, personal communication). In contrast to Australia, the human development index (HDI) of PNG is low (0.505, ranked 158th in the world). Therefore, access to education and health services in rural regions are limited due to poor infrastructure and lack of resources. However, residents in South Fly have access to emergency health care in the Torres Strait (24).

### Selection of Informants

Informants were selected purposively to obtain comprehensive information from a range of stakeholder groups. Informants were all older than 18 years. The stakeholder groups included the following:

- traditional leaders and elders,
- councilors (including divisional managers),
- veterinarians, environmental health workers (EHWs), and animal management workers (AMWs),
- biosecurity officers,
- health workers (HWs), including nurses and pharmacists,
- teachers,
- community residents.

Other information and perspectives were gained from people who were selected opportunistically during field-trips; for example, aircraft pilots, Animal Management in Rural and Remote Indigenous Communities employees,^2^ and hospitality, building, and retail industry workers. Direct observations were also made during visits to communities regarding the general level of dog health.

### Interview Structure and Data Collection

Data collection took place between February and June 2016. Informants were interviewed in their homes or workplaces. Interviews were semi-structured, so that informants could talk freely and about topics that they considered important. This flexibility also allowed researchers to investigate themes more thoroughly, dependent on informant experiences and perspectives. Interviews were guided by selecting open-ended questions relevant to stakeholder groups from a list of topics (Table 1). Time for each interview was determined by the informants’ willingness to talk and was not limited by the researchers. Researchers were accompanied by community members who translated interviews when informants preferred to talk in languages other than English. Informants were offered parasiticides for their dogs, to thank them for participation in the study.

**Table 1:** **Topics and example questions used in semi-structured interviews in a study to identify factors that will influence the acceptability and value of community-based rabies surveillance in Northern Australia and Papua New Guinea (PNG)**.

Topic 1: effective modes of communication within and between communities. If there is an important announcement for the community, how do you make sure that everybody knows about it? How do people communicate between communities? How do you think we could get people to tell us concerns about their dogs?
Topic 2: motivation and barriers to report concerns about dogs to other community members or organizations. What is the value of dogs to individuals; for example, is there connection to dogs through culture and what is the purpose of keeping dogs? What would motivate you to report health concerns about your dog? Why wouldn’t you report health concerns about your dog?
Topic 3: levels of expectations about dog health. What diseases do you see in your dogs? Informants’ descriptions of clinical signs associated with endemic differential diagnoses of rabies (for example, snake envenomation and cane-toad intoxication), Awareness and level of concern about dog health, Current level and perception of need for veterinary care in the community.
Topic 4: awareness of biosecurity regulations and rabies. Have you heard of rabies? When you travel, are you aware of quarantine zones and biosecurity requirements? This question was specific to PNG, Torres Strait and Northern Peninsula Area residents.
Topic 5: human health and perspectives on community dogs by health workers. Do residents seek treatment for bite wounds at health centers? Do you like community dogs and acknowledge them when they come with their owners to the clinic, or you visit patients in their homes? What do you think is the most appropriate way to deliver health messages to community members?

### Data Analysis

Interview transcripts and opportunistically collected information and perspectives were collated using qualitative data analysis software (NVivo; QSR International Pty Ltd. Version 11, 2015). Themes within the topics in Table 1 were identified in transcripts and triangulated between communities and regions and with peer-reviewed literature to assess consistency, divergence, and validity of themes.

## Results

Thirty-two semi-structured interviews were conducted with 64 informants (Table 2), of whom 73% were Aboriginal or Torres Strait Islander peoples of origin within the study regions. All informants were resident in the study regions. Interview duration ranged from 20 min to 1.5 h. All informants were willing to talk about their personal and workplace experiences related to dogs.

**Table 2:** **Number of semi-structured interviews and informants in a study to identify factors that will influence the acceptability and value of community-based rabies surveillance in Northern Australia and PNG**.

	East Arnhem	NPA	Torres Strait	PNG
Community elders, leaders, residents	6/6	2/4	3/3	9
Veterinarians, animal and environmental health workers, rangers, biosecurity officers, council workers	3/8	2/6	5/11	0
Human health workers, teachers	2/3	3/9	3/5	0
Total interviews/informants	11/17	7/19	11/19	3/9

*NPA, Northern Peninsula Area; PNG, Papua New Guinea*.

The strongest theme for effective modes of communication both within and between communities was verbal communication. This theme was consistent among informants from all study regions and backgrounds. Informants commonly described this as “passing on the message.” Verbal communication from elders, village leaders, or councilors to other community members and children was considered particularly important. Preferred modes of verbal communication for dissemination of information between and within communities included direct conversation, listening to the radio, or attending community meetings. Direct communication was the preferred method to inform other people about concerns such as poor dog health. In East Arnhem, informants were most likely to communicate concerns to elders and in other regions in this study, informants stated that they would speak to authorities, including biosecurity officers and AMWs or EHWs. Social media was also mentioned as potentially useful in some regions (East Arnhem) and with some participant groups (young adults and school children in the NPA and Torres Strait), highlighting that effective modes of communication should be re-assessed as the use of social media develops in these regions. Pictures were also thought to be a potentially useful method to disseminate information, but delivery of messages *via* written media (for example, leaflets and posters in health clinics) appeared less likely to be effective. Written media were not mentioned by Indigenous informants, and HWs said that leaflets and posters were largely ignored by patients and community members. In general, education about animal health and biosecurity was difficult to include in primary schools (teachers explained that there is insufficient space for these topics unless they support activities within the existing curriculum), and students in high school do not value agriculture-based classes. Informants also stated that information about rabies surveillance should be delivered in local languages because English is not the first language of many residents. For example, most Torres Strait Islanders speak at least three languages (their local Indigenous language, Torres Strait Creole, and English). Excerpts from interview transcripts relevant to the theme of communication are shown in Box 1.

Box 1 **Excerpts from interview transcripts about modes of communication in a study to identify factors that will influence the acceptability and value of community-based rabies surveillance in Northern Australia and Papua New Guinea.**

Community elder: “Everybody listens to the radio.”

Community elder: “We will help spread the message about the disease. On radio also.”

Community elder: “It is easy to teach the children, to pass on the stories.”

Community elder: “The people, they listen to Yolngu [people of East Arnhem] radio.”

Community member: “Talking to people—by visits”

Community elder: “Information needs to be passed from elders and parents through the kinship system.”

Health worker: “Radio is best for messaging… Everybody listens to [local radio] throughout Torres Strait and NPA.”

Councillor: “We have public meetings about everything. Some people… they can get very intense.”

PNG village leader: “We have meetings with the Torres Strait councillors… here and over there… to come up with solutions for our problems.”

Health worker: “We are kinaesthetic learners, so pictures are really good.”

Community elder: “I’m on Facebook, I like to stickybeak… some people poke me, but I keep quiet.”

Teacher: “Agriculture is a dying subject… kids don’t want to go outside.”

Dog value was a potential motivator to report health concerns about dogs. In the NPA and PNG, dogs are valued for hunting because they provide an important source of cheap protein (feral pigs, deer, or cattle). In East Arnhem, the cultural value of dogs was strong—informants described how harm to dogs can cause tangible pain to individuals for whom the dog is their totem. Informants in this region often noted that dogs are part of their family; researchers observed that some dogs had “skin names” and were included in the kinship system. Although the cultural value of dogs was more abstract in other regions, dogs were totems for individuals throughout the study regions (including PNG). In some circumstances, dogs also had economic value apart from hunting. For example, East Arnhem informants described payments to a dog’s owner if a dog is killed because the dog can no longer protect the owner. Payments could also be made to custodians of dog dreaming if a dog is harmed so that pain to ancestors and people connected to dogs through their totem is avoided. Dogs were also valued as companions in all regions and to enhance the social status of the owner in some regions. This was particularly apparent in the Torres Strait, where residents were sometimes keen to acquire particular breeds of dog from the Australian mainland.

Informants said that the lack of veterinary services in most regions was the greatest barrier to reporting health concerns in dogs. This theme was consistent between all informants and regions in this study. In the context of this study, a veterinary service refers to availability of a registered veterinarian who is able to examine animals, provide diagnoses, and prescribe treatments. A veterinary service is available in East Arnhem, but comprehensive coverage is limited due to the size of this region (35,000 km^2^) and the workforce available: one veterinarian based in a clinic in Yirrkala during the study period. In the Torres Strait, a veterinarian visits the region biannually and provides basic services such as dog population control. Veterinary services were not available in PNG and the NPA during and at least 2 years prior to the study period. Therefore, animal health care is generally limited to services provided by AMWs and EHWs, which include supply of parasiticides and disposal of dead dogs in some regions. Informants were aware of potential zoonotic disease transmission (another potential motivator)—for example, dogs with skin disease such as mange and ticks were perceived as a risk to family health. Throughout all regions, informants stated that parasitic skin disease in dogs was common and wanted parasiticides for their dogs. In Northern Australian regions, cane-toad poisoning and snake envenomation (both differential diagnoses for rabies) are sufficiently common in dogs that informants could describe clinical signs. Informants explained that there was no incentive to report these concerns because treatment was not available. This led to acceptance that unhealthy dogs are a normal, daily occurrence. Consistent with this theme, researchers observed that dogs appeared generally healthier in regions in which veterinary services were available intermittently (Torres Strait islands) or continuously (Yirrkala, East Arnhem).

Other themes about barriers to reporting were region-specific or less common. Lack of trust of authorities and fear of shame or recrimination within the community were both barriers to reporting concerns about dogs in East Arnhem. This was attributed to historical experiences such as inhumane dog control. Throughout all regions in the study there was also an apparent lack of concern for dog welfare by some community members, attributed to insufficient connection to culture, and therefore, lack of knowledge that dogs are part of family. Excerpts from interview transcripts relevant to motivation and barriers to reporting health concerns in dogs and level of expectations about dog health are shown in Box 2.

Box 2 **Excerpts from interview transcripts about motivation and barriers to reporting health concerns in dogs, and level of expectations about dog health in a study to identify factors that will influence the acceptability and value of community-based rabies surveillance in Northern Australia and Papua New Guinea.**

Community elder: “This is dingo land. Dog is not alone from humans. Through the spirit, the journey, we feel them close…”

PNG leader: “I have two dogs (two females), with six babies… I like them. My totem is dog.”

Councillor: “You can’t restrict everybody to two dogs. Hunting is big here… they need more [than two] dogs…”

Health worker: “Dogs are a totem … here in the NPA, in Bamaga in particular, there is a high cohort of Umai [dog] totem. This comes from Saibai.”

PNG community leader: “We look after our dogs like we look after our children because they also provide food for our children… dogs are valuable”

Councillor: “If you cull dogs, people get upset. Not because of cultural beliefs, but because they are attached to their dog.”

Community elder: “If a dog gets sick you can put a blanket on it. There is nobody to help.”

Community elder: “We need more [veterinary] service as well as awareness of disease.”

Veterinarian: “We have to follow up cheeky [aggressive] dogs, but it can be difficult because people think we are going to take the dog away.”

Health worker: “People worry that the dogs will get blamed if they [the people] have skin problems. They hide the dogs.”

Community member: “She didn’t want the ticks on the verandah because of the children [so she threw the boiling water at the dog]… she didn’t realise it would be long-time pain.”

Community elder: On reasons for animal cruelty and neglect, “Important song-lines are not shared. The young people don’t know them.”

Indigenous informants were aware of the importance of biosecurity and stated that they complied with regulations when traveling between PNG, the Torres Strait and mainland Australia. Non-indigenous residents more commonly had either limited knowledge, lack of concern for regulations, or openly admitted non-compliance because the regulations were inconvenient. Although most people had heard of rabies, very few were aware of the risk of an incursion due to entry of infected dogs from endemic regions or the zoonotic potential and impact of rabies in humans. A few people recalled a picture of a rabid dog on biosecurity information for passengers in ferries and aircraft in the region. Human HWs were aware of rabies, but it was not something that they considered in their daily work—they were occupied with current health risks that included multi-drug resistant tuberculosis (in PNG residents seeking healthcare in Torres Strait Island health clinics) and the recent outbreak of Dengue Fever in the Torres Strait region.

Health workers (most of whom were non-indigenous) estimated that the majority of people with dog-bite wounds would seek medical attention because wound infections are viewed as a common cause of more serious conditions such as septicemia. Consistent with this opinion, the few informants who had experienced dog-bite wounds had attended a health clinic for treatment. HWs’ perceptions and level of acceptance of dogs in community were variable; some recognized dogs as part of the family and acknowledged and treated unhealthy dogs. Other HWs felt threatened by community dogs in public places, were unaware that free-roaming dogs in Northern Australia are owned, were not interested in dog health and perceived unhealthy animals as an indicator of lack of concern for dog welfare by Indigenous community residents. This variability was also found in other groups of non-indigenous informants such as teachers and hospitality industry workers. Excerpts from interview transcripts relevant to awareness of biosecurity regulations, rabies, and the interface between human and dog health are shown in Box 3.

Box 3 **Excerpts from interview transcripts about awareness of biosecurity regulations, rabies and the interface between human and dog health in a study to identify factors that will influence the acceptability and value of community-based rabies surveillance in Northern Australia and Papua New Guinea.**

Health worker: “So many times, people ask me to help with their dog… Other nurses say they don’t want to have anything to do with the animals, but that affects their relationship with the families, because then they are not part of the holistic approach. Dogs are part of the family.”

Health worker: “Dogs should be removed from the clinic waiting area… people should only be allowed one dog per household by law.”

Health worker; “it is important to empower people through self-management of health… but this is a challenging approach for some HWs in a system which has traditionally advocated a custodial approach to Indigenous health.”

Health worker; “We don’t see many issues from dogs. Bites are rare… we see a few cat scratches on children.”

Councillor: On the value of dogs in the community in comparison to other community issues, “Dogs are still important… it’s all interrelated.”

Non-indigenous community member: “When I visit my son on TI we take the dogs. We don’t go through quarantine on the way back, but it’s OK—the dogs don’t go anywhere, they don’t meet other dogs.”

PNG community leader: “We have our control measures making sure we don’t violate our [quarantine] guidelines and we maintain our treaty agreement….”

## Discussion

Effective communication is vital for community-based surveillance; participants need to understand reporting requirements and reports need to be collected in a timely manner for analysis. The most consistent theme for this topic throughout all study regions was the importance of verbal communication and informants in this study commonly talked of “passing on the message” about rabies. This is consistent with the cultural background of Indigenous people in Northern Australia and PNG. Aboriginal and Torres Strait Islander lore has been passed down generations for tens of thousands of years by elders, using stories, ceremonies, dance, music, and art.^3^ Story-telling (such as “sing-sings”) is also a fundamental feature in PNG culture. For example, Mercer et al. (25) found that the impacts of a tsunami in 1930 on the north coast of mainland PNG were mitigated because residents knew stories—which had been passed down verbally through generations—that described warning signs and evacuation to higher ground. In contrast to Western culture in which information is commonly disseminated in written form and directed from authorities to children, we suggest that messages to enhance rabies surveillance in Northern Australia and PNG should be verbal and use traditional communication channels by engaging elders and councils to “pass on the message.” These strategies also build on connection to culture and encourage traditional values such as care for dogs in community. Messages should be delivered in local languages. This has also been recognized as important for effective communication in sectors other than biosecurity. For example, the Queensland Government Department of Health provides an interpreter service which includes Tok Pisin, the PNG equivalent of Torres Strait Creole.

Although several modes of verbal communication were identified *via* which messages to enhance rabies surveillance might be delivered, modes to report clinical signs were limited to direct communication. In addition, the preferred routes for reporting varied between regions. In the Torres Strait and NPA, informants stated that they would report concerns to AMWs or biosecurity officers; in these regions, there is comprehensive coverage by these groups, both geographically and temporally, and they are already familiar with the importance of animal health. However, in Western Province PNG, there are no animal health services, and in East Arnhem there is mistrust of authorities due to previous experiences of inappropriate responses to perceived animal health issues. This poses challenges for collection of high quality surveillance data over such a large region because the structure of data collection might need to be regionally customized. In regions such as PNG and East Arnhem, it is likely that community elders will need to be engaged to encourage reporting.

This study also highlighted other important challenges and barriers to rabies surveillance in Northern Australia and Western Province, PNG. We found that dogs are valued for a variety of reasons which can be intangible (for example, connection to culture and companionship) or tangible (for example, their value as hunting animals). This is consistent with the findings of Constable et al. (6). Although the value of dogs is a potential motivator to report health concerns about dogs, the lack of veterinary services was a major barrier. Without direct health benefits to dogs, reporting of clinical signs is unlikely to be sustainable. This is illustrated by the acceptance of the lower level of general dog health in communities in which veterinary services were unavailable. The importance of a returned value (perceived benefit) to those that report in surveillance systems has been highlighted previously by Syibli et al. (26) and illustrated by the development of a mobile phone reporting system in Indonesia.^4^ Primarily, this system provides benefit to those involved directly in animal health (for example, farmers and veterinarians) and has a secondary purpose as a tool for syndromic surveillance. Preliminary reports suggest that the value of animal health care is a sufficient incentive to report clinical signs and achieve syndromic surveillance with good temporal and geographic coverage in some Indonesian regions (27). In addition to the lack of veterinary services as a barrier to reporting clinical signs, the specificity of clinical signs for rabies is low due to common endemic syndromes with similar clinical signs. As well as likely reducing sustainability through false-positive incursion alerts, this also has implications for messaging about surveillance; community-wide surveillance for rabies-associated clinical signs should be carefully considered so as not to induce panic about dogs with clinical signs which currently are more likely to indicate non-zoonotic syndromes, such as snake envenomation or cane-toad poisoning. Overall, we believe that unless veterinary services can be improved consistently throughout this region, community-wide surveillance of dog mortality and training of selected community leaders and workers to identify clinical signs is likely to be more acceptable and sustainable than community-wide surveillance for clinical signs consistent with rabies. This level of syndromic rabies surveillance has a number of advantages: it builds on the already valued service provided by EHWs and AMWs in some regions to dispose of dead dogs and it is 100% sensitive for rabies (all rabies-infected dogs die). In addition, messages to report dog mortality are unlikely to induce the same fear that reporting suspect cases of rabies would. Although mortality is not specific to rabies, increased incidence of dog mortality is a useful indicator of other important diseases such as distemper (endemic) and screw-worm fly (exotic).

We also identified potential gaps in community engagement for rabies surveillance. The Torres Strait is a cultural and geographic interface between PNG and mainland Australia, and compliance with biosecurity regulations is important to maintain freedom of movement for traditional purposes, while protecting human, animal, and plant health in this region (The Torres Strait Treaty).^5^ We found that the level of awareness of the importance of these regulations was variable among informants, particularly non-indigenous residents such as HWs, teachers, and other industry workers. Understanding the value and acceptability of community-based rabies surveillance to engage non-indigenous residents was not an objective of this study. In particular, HWs and teachers are positioned to provide valuable surveillance of both animal and human health in these regions—there is an extensive network of human health care facilities and schools in Indigenous communities throughout Northern Australia, and residents in coastal Western Province PNG have access to emergency healthcare in the Torres Strait. However, informants in these groups were also sometimes unaware of the value of dogs to communities or the risk of rabies to the region and therefore, dismissed the importance of community dog health. Further studies to investigate methods to improve engagement of non-indigenous community residents in biosecurity practices and encourage a One Health approach to community health are worth pursuing. In addition to enhancing the sustainability and community-wide coverage of rabies surveillance, improved understanding of the value of dogs and the way in which they are kept in communities is also likely to promote trust between Indigenous community members and those of non-indigenous origin in regions such as East Arnhem. Among other benefits, this could also enhance reporting for community-based rabies surveillance.

Qualitative research asks questions that are fundamentally different to those studied in quantitative research (13, 14) to document and explain a range of views, needs, values, practices, and beliefs. For example, a qualitative study might investigate why people make decisions to report health concerns, whereas a quantitative study might investigate the proportion of people that report health concerns. Although lack of quantitative data obviously precludes evaluation of precision, assessments can be made concerning the internal and external validity of qualitative study findings. Limitations of the current study could include selection bias of communities and informants and information error due to interpretive bias by the researchers during thematic analysis of transcripts. However, the themes of the importance of verbal communication and traditional communication channels, the high value of dogs to Indigenous community residents, and the lack of veterinary services as a barrier to reporting were consistent throughout the study region across informants with a range of backgrounds. In addition, these themes are consistent with other research and Indigenous Australian and PNG history; this increases the generalizability of these findings across Northern Australia and Western Province, PNG. Divergent themes—for example, cruelty to dogs associated with lack of connection to culture in some communities or the use of social media for communication—are less generalizable. Quantitative studies could be used to investigate the importance of these themes, and methods from participatory epidemiology (which also include semi-structured interviews) could be used to determine the frequency of these activities relative to actions that promote dog welfare in communities (17). Perhaps a more important limitation of this study was the assessment of acceptability and value of community-based rabies surveillance out of context of competing community concerns. For example, health outcomes are poorer, levels of education achieved are lower, and a greater proportion of people serve custodial sentences in Aboriginal and Torres Strait Islander communities than other communities in Australia (28, 29), and the HDI in PNG is low (0.505).^6^ Despite the improved sustainability of community-based surveillance that could be achieved using the findings of the current study, surveillance for animal health might ultimately be of insufficient value in comparison to other community concerns to achieve timely detection of a rabies incursion in these regions.

Consistent with previous studies (21–23), the qualitative methods used in this study provided important insights about the acceptability and value of community-based rabies surveillance in Indigenous communities in Northern Australia and Western Province, PNG. The findings of this study will inform design of communications materials—such as radio stories—to enhance appropriate syndromic (for example, dog mortality), community-based rabies surveillance using traditional communications channels. The qualitative methods used in this study complemented the quantitative methods used in previous studies in this region that identified the comparative risk of regions in Northern Australia and PNG (4, 30). Together, the findings of these studies can be used to design sustainable surveillance strategies targeted to high-risk regions and increase the probability of effective surveillance to limit outbreak size and detect disease in dogs before a human death occurs. However, it should be noted that sustainable surveillance will also depend on the integration of surveillance activities within the context of competing concerns in communities. As described by informants, community health requires a holistic approach because all aspects of community life are interrelated.

## Ethics Statement

This study was carried out in accordance with the recommendations of the Australian National Health and Medical Research Council’s National Statement on Ethical Conduct in Human Research, the Human Research Ethics Committees of the Northern Territory Department of Health and The University of Sydney with informed verbal consent from all subjects. The protocol was approved by the Human Research Ethics Committees of the Northern Territory Department of Health and The University of Sydney (reference numbers 2016-2606 and 2016/192, respectively).

## Author Contributions

All authors contributed to the conception and design of this study and acquisition of data. VB was responsible for analysis and interpretation of the data. All authors contributed to critical revision of this manuscript and agreed to be accountable for all aspects of this study.

## Conflict of Interest Statement

The authors declare that the research was conducted in the absence of any commercial or financial relationships that could be construed as a potential conflict of interest.

## Footnotes

^1^http://www.abs.gov.au/census.

^2^http://www.amrric.org, accessed 13.09.16.

^3^http://aiatsis.gov.au/, accessed 20.09.16.

^4^http://wiki.isikhnas.com/, accessed 20.09.16.

^5^http://dfat.gov.au/geo/torres-strait/Pages/the-torres-strait-treaty.aspx, accessed 20.09.16.

^6^http://hdr.undp.org/en/countries/profiles/PNG, accessed 20.09.16.

## References

[1] Amaral AC Ward MP Freitas JDC. Estimation of roaming dog populations in Timor Leste. Prev Vet Med (2014) 113(4):608–13. 10.1016/j.prevetmed.2013.11.012 24360218

[2] Ward MP Hernandez-Jover M. A generic rabies risk assessment tool to support surveillance. Prev Vet Med (2015) 120(1):4–11. 10.1016/j.prevetmed.2014.11.005 25466214

[3] Ward MP. Rabies in the Dutch East Indies a century ago – a spatio-temporal case study in disease emergence. Prev Vet Med (2014) 114(1):11–20. 10.1016/j.prevetmed.2014.01.009 24485277

[4] Brookes VJ Ward MP. Expert opinion to identify high-risk entry routes of canine rabies into Papua New Guinea. Zoonoses Public Health (2016). 10.1111/zph.12284 27362859

[5] Hampson K Coudeville L Lembo T Sambo M Kieffer A Attlan M Estimating the global burden of endemic canine rabies. PLoS Negl Trop Dis (2015) 9(4):e0003709 10.1371/journal.pntd.0003709 25881058 PMC4400070

[6] Constable S Dixon R Dixon R For the love of dog: the human-dog bond in rural and remote Australian indigenous communities. Anthrozoos (2010) 23(4):337–49. 10.2752/175303710x12750451259336

[7] Dwyer PD Minnegal M Wild dogs and village dogs in New Guinea: were they different? Aust Mammal (2016) 38(1):1–11. 10.1071/am15011

[8] Tenzin T Ward MP. Review of rabies epidemiology and control in South, South East and East Asia: past, present and prospects for elimination. Zoonoses Public Health (2012) 59(7):451–67. 10.1111/j.1863-2378.2012.01489.x 23180493

[9] Townsend SE Sumantra IP Pudjiatmoko Bagus GN Brum E Cleaveland S Designing programs for eliminating canine rabies from islands: Bali, Indonesia as a case study. PLoS Negl Trop Dis (2013) 7(8):e2372. 10.1371/journal.pntd.0002372 23991233 PMC3749988

[10] Anonymous. Aiming for elimination of dog-mediated human rabies cases by 2030. Vet Rec (2016) 178(4):86–7. 10.1136/vr.i51 26795858

[11] ProMED-mail. *Rabies* – Malaysia (Perlis): Canine, OIE, Archive Number: 20150825.3599775, Published Date: 2015-08-25 06:03:38 (2015). Contract No.: 20150825.3599775.

[12] Kitala PM McDermott JJ Kyule MN Gathuma JM Community-based active surveillance for rabies in Machakos district, Kenya. Prev Vet Med (2000) 44(1–2):73–85. 10.1016/s0167-5877(99)00114-2 10727745

[13] Christley RM Perkins E Researching hard to reach areas of knowledge: qualitative research in veterinary science. Equine Vet J (2010) 42(4):285–6. 10.1111/j.2042-3306.2010.00074.x 20525043

[14] Britten N Jones R Murphy E Stacy R Qualitative research methods in general-practice and primary-care. Fam Pract (1995) 12(1):104–14. 10.1093/fampra/12.1.104 7665030

[15] Pope C Mays N Reaching the parts that other methods cannot reach – an introduction to qualitative methods in health and health-services research. Br Med J (1995) 311(6996):42–5. 7613329 10.1136/bmj.311.6996.42 PMC2550091

[16] Pope C Mays N Critical reflections on the rise of qualitative research in health services research. J Epidemiol Community Health (2008) 62:A1–36.18757454

[17] Catley A Alders RG Wood JL. Participatory epidemiology: approaches, methods, experiences. Vet J (2012) 191(2):151–60. 10.1016/j.tvjl.2011.03.010 21856195

[18] Birhanu A Worku T Benti D. Investigation of major cattle production constraints in Kembata Tambaro zone of Southern Ethiopia using participatory epidemiology methods. Trop Anim Health Prod (2016) 48(1):109–15. 10.1007/s11250-015-0928-y 26477032

[19] Byaruhanga C Oosthuizen MC Collins NE Knobel D Using participatory epidemiology to investigate management options and relative importance of tick-borne diseases amongst transhumant zebu cattle in Karamoja region, Uganda. Prev Vet Med (2015) 122(3):287–97. 10.1016/j.prevetmed.2015.10.011 26527312

[20] Kabaka W Gitau GK Mariner J Abudiku N The use of participatory epidemiology to determine the prevalence rate and economic impacts of PPR and CCPP in Turkana county of Kenya. Bull Anim Health Prod Afr (2012) 60(3):241–50.

[21] Calba C Antoine-Moussiaux N Charrier F Hendrikx P Saegerman C Peyre M Applying participatory approaches in the evaluation of surveillance systems: a pilot study on African swine fever surveillance in Corsica. Prev Vet Med (2015) 122(4):389–98. 10.1016/j.prevetmed.2015.10.001 26489602

[22] Calba C Goutard FL Vanholme L Antoine-Moussiaux N Hendrikx P Saegerman C The added-value of using participatory approaches to assess the acceptability of surveillance systems: the case of bovine tuberculosis in Belgium. PLoS One (2016) 11(7):e0159041 10.1371/journal.pone.0159041 27462705 PMC4962975

[23] Schulz K Calba C Peyre M Staubach C Conraths FJ Hunters’ acceptability of the surveillance system and alternative surveillance strategies for classical swine fever in wild boar – a participatory approach. BMC Vet Res (2016) 12(1):187 10.1186/s12917-016-0822-5 27601050 PMC5012045

[24] Foreign Affairs Defence and Trade References Committee. The Torres Strait: Bridge and Border. Canberra: Commonwealth of Australia (2010). p. 978–971.

[25] Mercer J Gaillard JC Crowley K Shannon R Alexander B Day S Culture and disaster risk reduction: lessons and opportunities. Environ Hazards Hum Policy Dimens (2012) 11(2):74–95. 10.1080/17477891.2011.609876

[26] Syibli M Nurtanto S Yulianti S Yohana CK Cameron AR Muljono AT The power of one: realising the dream of an integrated animal health information system in Indonesia. International Conference on Animal Health Surveillance 2 (ICAHS2) Havana, Cuba (2014).

[27] Happold J Tackling emerging disease threats at source: the Australian government’s partnerships with Indonesia, Timor Leste and Papua New Guinea. Australian and New Zealand College of Veterinary Scientists, Annual Conference Gold Coast (2016).

[28] Department of the Prime Minister and Cabinet. Closing the Gap: Prime Minister’s Report 2016 Canberra: Commonwealth of Australia (2016). Available from: http://closingthegap.dpmc.gov.au/assets/pdfs/closing_the_gap_report_2016.pdf

[29] House of Representatives – Standing Committee on Aboriginal and Torres Strait Islander Affairs. Doing Time – Time for Doing: Indigenous Youth in the Criminal Justice System. Canberra: Commonwealth of Australia (2011). Available from: http://www.aph.gov.au/parliamentary_business/committees/house_of_representatives_committees?url=atsia/sentencing/report.htm

[30] Brookes VJ Ward MP Risk Assessment: The Entry of Canine-Rabies into Papua New Guinea via Land and Sea Routes. Gold Coast, QLD: Australian and New Zealand College of Veterinary Scientists, Science Week (2016).

